# Distinct immune properties of the N- and C-termini of the immunosuppressive domain of Ebola virus glycoprotein

**DOI:** 10.1128/mbio.02278-25

**Published:** 2025-10-09

**Authors:** Mathieu Iampietro, Sivakumar Periasamy, Philipp A. Ilinykh, Yuan Qiu, Yakun Liu, Abhinit Nagar, Bin Gong, Alexander Bukreyev

**Affiliations:** 1 Department of Pathology, University of Texas Medical Branch 198642, Galveston, Texas, USA; 2 Galveston National Laboratory, The University of Texas Medical Branch 12338, Galveston, Texas, USA; 3 Centre International de Recherche en Infectiologie (CIRI), University of Lyon, Inserm, CNRS, Université Claude Bernard Lyon 1, Ecole Normale Supérieure de Lyon 133614 , Lyon, France; 4 Luminex Corporation 17737 , Austin, Texas, USA; 5 Department of Microbiology & Immunology, The University of Texas Medical Branch 12338, Galveston, Texas, USA; Tsinghua University, Beijing, China

**Keywords:** ebola virus, cytokine, immunosuppression, inflammation

## Abstract

Ebola virus (EBOV) causes a severe human disease with high lethality. Pathogenesis of EBOV disease is characterized by a paradoxical combination of hyperinflammation and immunosuppression. EBOV has a single envelope glycoprotein (GP), which is a type I transmembrane protein with strong immunomodulatory effects. GP contains a conserved immunosuppressive domain (ISD) with a high similarity to ISDs of envelope proteins retroviruses. To investigate the effects of ISD, a set of 17 EBOV viral-like particle (VLP) constructs containing the entire EBOV nucleoprotein, VP40, and GP with single alanine or glycine substitutions in each position of ISD was generated and tested in human peripheral blood mononuclear cells (PBMCs). Wild-type VLPs induced inflammatory responses; however, when added to pre-stimulated cells, they reduced inflammation, thus exerting immunosuppressive properties. Substitution of lysine at ISD position 5 (Lys-5) increased anti-inflammatory properties by reducing proliferative responses of VLPs and also reducing nuclear factor of activated T-cells 1 (NFAT1) and nuclear factor kappa-light-chain-enhancer of activated B cells (NF-κB) activation. In contrast, substitution of tryptophan at position 14 (Trp-14) increased both the pro-inflammatory and the proliferative responses and the adhesion of VLP-infected human monocytes to microvascular endothelial cells. Thus, the ISD N- and C-termini have pro- and anti-inflammatory properties, respectively, suggesting their unique implications in the EBOV pathogenesis. Furthermore, the immunomodulating effects of ISD were also mediated by shed GP, which is abundant in the medium. These data may be useful for the development of treatments for diseases caused by EBOV by targeting the ISD.

**IMPORTANCE:**

Our data suggest that the ISD N-terminus plays a role in activating immune cells and pro-inflammatory response. In contrast, the C-terminus of ISD downregulates the pro-inflammatory response through the reduction of NF-kB and NFAT activities. The data also show that EBOV GP increases the adhesion of monocytes to endothelial cells, and the effect is inhibited by the ISD C-terminus. Moreover, the data demonstrate that the immunomodulating effects of ISD are mediated not only by the virus-associated GP but also by the shed GP, which is abundant in the medium. Pathogenesis of the disease caused by EBOV is characterized by hyperinflammation and some features of immunosuppression, which could in part be affected by the complex effects of the ISD. These data indicate that targeting the ISD may be considered for the development of treatments for the disease caused by EBOV.

## INTRODUCTION

Ebola virus (EBOV) causes a highly lethal human disease, characterized by a fatality rate ranging from 45% to 90% (1–3). EBOV infects a wide range of immune cells and causes excessive immunomodulation involving both pro- and anti-inflammatory properties, leading to dysregulated and toxic immune responses (1–4).

EBOV belongs to the *Filoviridae* family and has a single-stranded non-segmented negative-sense RNA genome encoding seven major genes located in a linear order (5). The gene number four produces two major proteins: a 676-amino acid-long type 1 transmembrane glycoprotein (GP), which is anchored in the viral membrane, and a 364-amino acid-long soluble glycoprotein (sGP). GP is produced from mRNA undergoing co-transcriptional editing, whereas sGP is produced from its unedited version (6–8). GP is post-translationally cleaved by the host cellular protease furin and is displayed on the virion’s surface as two subunits, GP1 and GP2, linked by disulfide bonds (9). EBOV GP1 is highly glycosylated with N-linked and O-linked glycans, whereas GP2 contains mostly N-linked glycans (10). In addition, shed GP, which represents soluble full-length GP ectodomain resulting from proteolysis of GP by cellular tumor necrosis factor α-converting enzyme (TACE), is also present in the extracellular compartment (11).

EBOV GP mediates viral attachment, fusion to the membrane, and subsequent cellular entry (4, 11, 12). Furthermore, GP activates dendritic cells, macrophages, and T-cells through binding to toll-like receptor 4 (TLR4) (12–14). In addition, GP is implicated as a main factor of virulence contributing to vascular cell toxicity and injury that underlie the phenomenon of hemorrhagic fever occurring during EBOV infection (15, 16). Importantly, the interaction of viral GP with immune cell populations leads to dysregulation of the immune response during EBOV disease (4, 14). One of the viral factors involved in modulating the immune response could be the immunosuppressive domain (ISD) located in GP2 (17). EBOV GP ISD represents a sequence of 17 amino acids (a.a.) initially identified in the ectodomains of retroviral envelope proteins (18) and demonstrated to possess immunosuppressive activity in multiple experimental systems (18–22). Subsequently, a similar ISD was identified in the GP ectodomain of Marburg virus, which is another filovirus (23). However, the biological effects of EBOV and Marburg virus ISD remain obscure. One report studying the GP crystal structure suggested that the EBOV ISD is unlikely to have an immunosuppressive effect (24). Another study reported that incubation of human peripheral blood mononuclear cells (PBMCs) with a 17-mer peptide mimicking ISD of EBOV or other filoviruses inhibits activation of CD4^+^ and CD8^+^ T-cells (25); however, peptides may not necessarily reproduce biological properties of ISD in the context of the full-length protein. Here, to test the effects of ISD with a more authentic system, we generated a set of EBOV viral-like particles (VLPs) and recombinant shed GP with single point mutations in amino acid residues constituting the ISD. We exposed human PBMCs to the mutated and non-mutated VLPs. We found that a mutation in Lys-5 (N-terminus) enhances the release of anti-inflammatory factors, whereas the mutation in Trp-14 (C-terminus) has opposite effects. Overall, the strikingly opposite effects of the N- and C-termini of ISD may have implications in EBOV pathogenesis.

## RESULTS

### EBOV GP triggers inflammation in non-stimulated cells but has immunosuppressive effects in pre-stimulated cells

**Fig 1:**
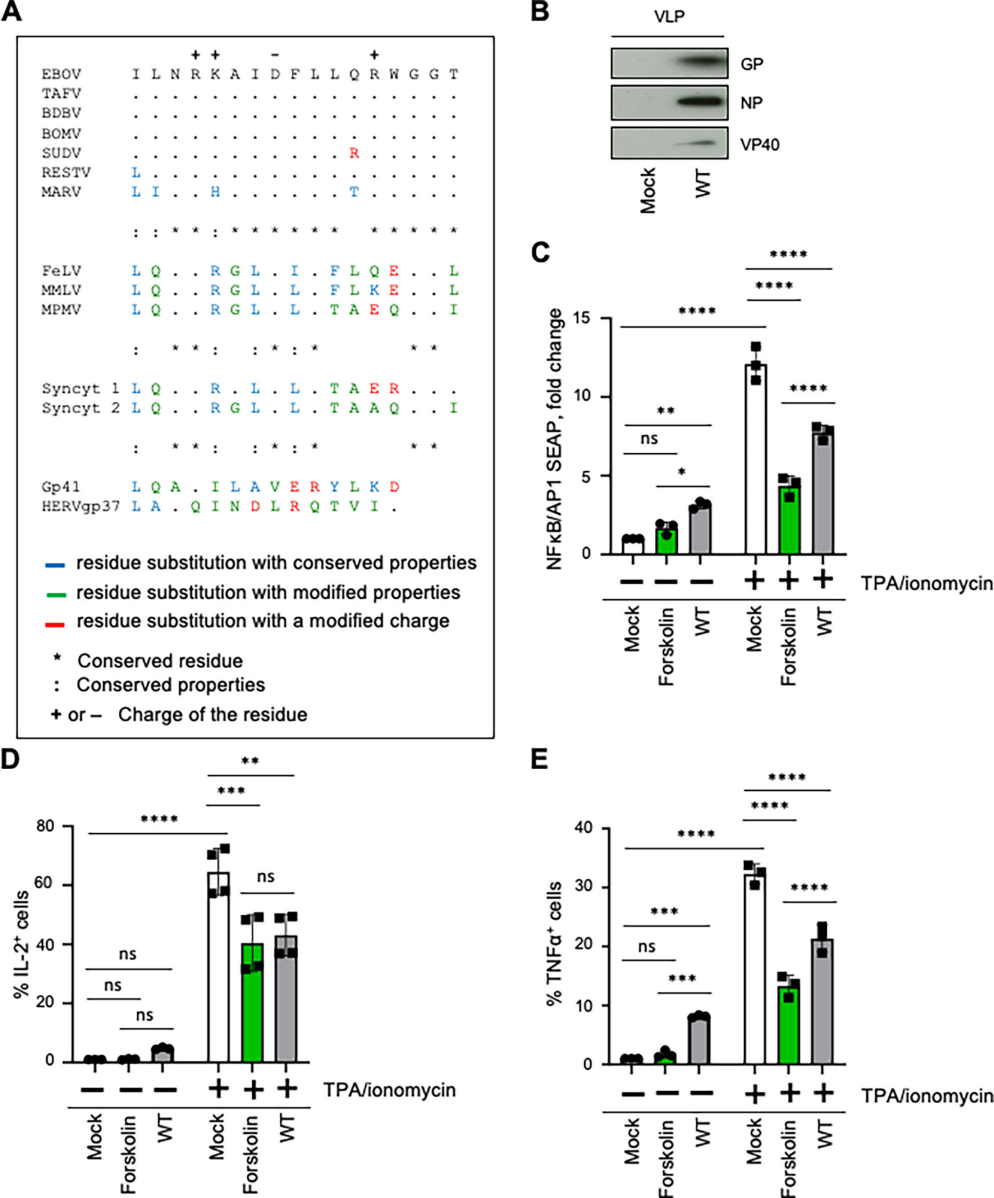
EBOV GP has an immunosuppressive effect in pre-stimulated cells. (**A**) Comparison of immunosuppressive domains on GP of selected filoviruses and endogenous or exogenous retroviral proteins. EBOV, Ebola virus; TAFV, Taï Forest virus; BDBV, Bundibugyo virus, BOMV, Bombali virus, SUDV, Sudan virus, RESTV, Reston virus, MARV, Marburg virus, FeLV, feline leukemia virus, MMLV, Moloney murine leukemia virus, MPMV, Mason-Pfizer monkey virus, Syncyt 1 (syncytin 1), human endogenous retrovirus-W, Syncyt 2 (syncytin 2), human endogenous retrovirus-FRD, gp41, human immunodeficiency virus 1, HERVgp37, human endogenous retrovirus K. (**B**) Western blot analysis of VLPs harvested from supernatants following transfection of 293T cells with plasmids encoding NP, VP40, and GP. (**C**) Effects of EBOV VLPs on NFκB/AP1 activity in pre-stimulated cells. THP-1 NFκB Xblue cells were stimulated or not with TPA and ionomycin and treated or mock-treated with forskolin or WT VLPs for 16 h. (**D, E**) Effects of EBOV VLPs on induction of IL-2 (**D**) or TNFα (**E**) by PBMCs. PBMCs were stimulated or not with TPA and ionomycin, treated or mock-treated with forskolin or WT VLPs for 16 h, and analyzed by flow cytometry. Data are represented as mean ± SEM from three independent experiments with different donors, with each donor sample analyzed in technical triplicate. All samples were analyzed using two-way ANOVA followed by a Tukey’s multiple comparison test: *P < 0.05, **P < 0.01; ***P < 0.001; ****P < 0.0001; ns, non-significant.

The EBOV GP ISD has various levels of amino acid similarity to ISD of other ebolaviruses and retroviruses (Fig. 1A). To verify that the ISD has a capacity to reduce inflammation, we generated VLPs by transfecting 293T cells with plasmids encoding VP40, NP, and WT GP. After 72 h post-transfection, the supernatants were harvested, and VLPs were purified by sucrose gradient centrifugation and analyzed for the incorporation of the EBOV proteins. Analysis of VLPs by western blotting demonstrated the presence of expected proteins (Fig. 1B). To evaluate the effect of ISD on inflammation, we tested the activities of key transcription factors, NF-κB and activator protein 1 (AP-1), which are involved in multiple biological and pathological processes, including inflammation (26), using THP-1 XBlue cells that allow monitoring their activation by expression of both NF-κB- and AP-1-inducible SEAP reporter gene. We exposed THP-1 XBlue cells to VLPs or medium from mock-transfected cells or pre-stimulated with 12-O-tetradecanoylphorbol-13-acetate (TPA) and ionomycin to induce a pro-inflammatory state (Fig. 1C). Although forskolin, used as a control for anti-inflammatory activity, inhibited inflammation in both culture environments, WT VLPs increased NF-κB and AP-1 activity in mock-treated cells (Fig. 1C left panel), whereas it impaired their activities when cells were previously treated with TPA/ionomycin (Fig. 1C right panel). We next repeated our experimental procedures using primary human PBMCs and found that both IL-2 (Fig. 1D) and TNFα levels (Fig. 1E) were slightly increased in mock-treated cells (Fig. 1D and E left panels), whereas their levels reduced following culture of WT VLPs in pre-activated cells (Fig. 1D and E right panels), confirming our previous results. Interestingly, these data describe complex effects of EBOV GP and the potential role of its ISD during EBOV immunopathogenesis, as it triggered inflammatory signatures in non-stimulated cells while significantly reducing inflammatory response in pre-activated cells.

### The N- and C-terminal amino acids of EBOV GP ISD have opposite immunomodulating effects

To better understand the immunomodulating effects of the EBOV GP ISD, we designed a set of VLPs including WT and 17 ISD single mutants, with an alanine (Ala) substitution in each residue of the 17-mer ISD sequence, except Ala in position 6, which was replaced with glycine (Gln) (Fig. S1A). This was followed by the generation of VLPs as described above. The mutant VLPs showed comparable expressions of GP, NP, and VP40 to that of WT VLP (Fig. S1B).

**Fig 2:**
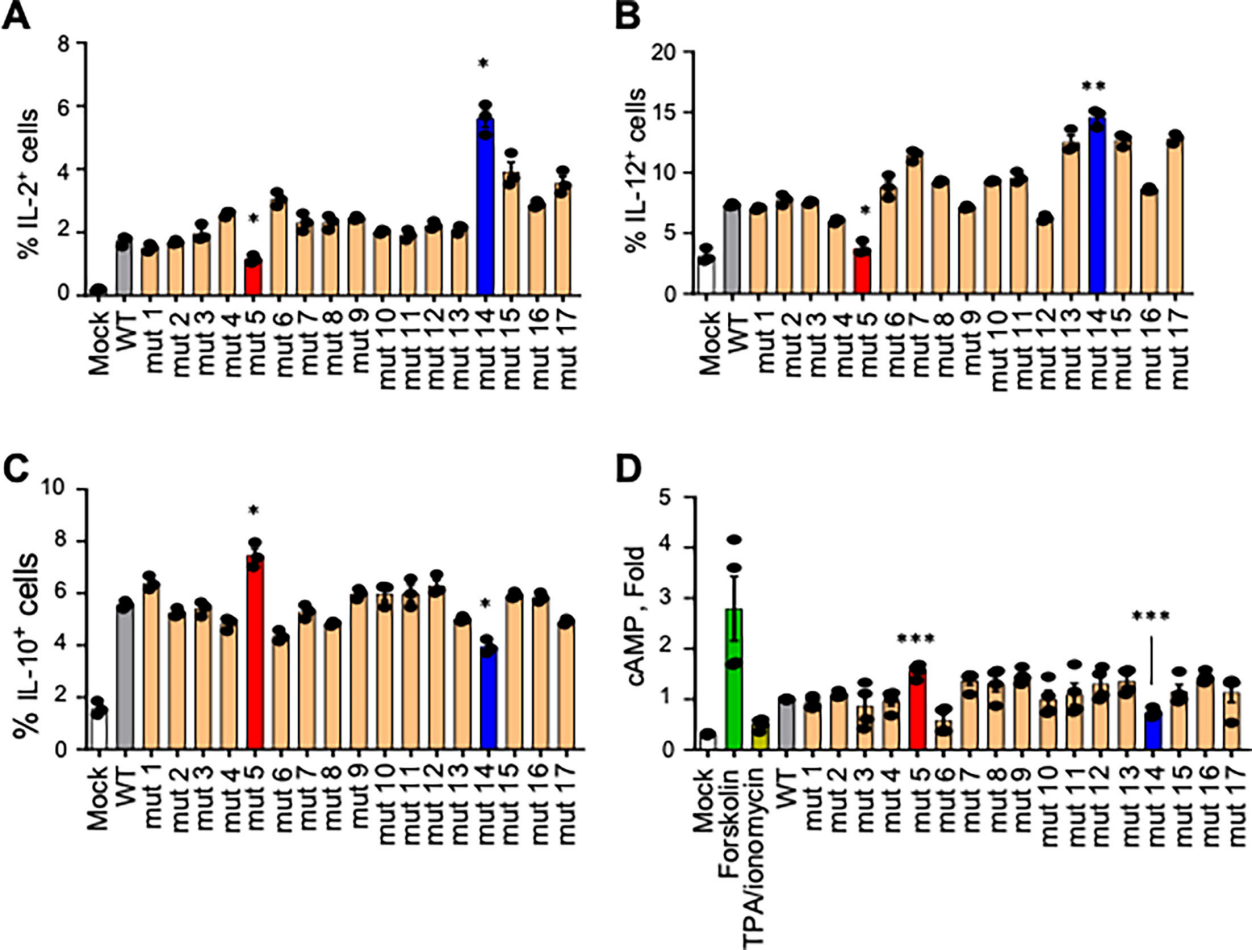
N- and C-termini of EBOV GP ISD have opposite effects on innate immune response in PBMCs. (**A to C**) Flow cytometry analysis for IL-2 (**A**), IL-12 (**B**), or IL-10 (**C**) in PBMCs stimulated with EBOV GP VLPs or their mutants. PBMCs were cultured with medium alone or with WT or each single mutant VLP of EBOV GP ISD (mut 1 to mut 17) for 24 h and analyzed by flow cytometry. (**D**) cAMP in PBMCs stimulated with WT or mutated EBOV GP VLPs for 24 h was determined by ELISA. Mean values ± SEM from three independent experiments with different donors, with each donor sample analyzed in technical triplicate. All samples were analyzed using one-way ANOVA followed by a Dunnett’s multiple comparison test; comparison of individual mutants vs WT VLPs: *P < 0.05, **P < 0.01; ***P < 0.001; ns, non-significant.

To test the effects of the ISD mutations on the expression of cytokines, human PBMCs were exposed to WT or mutant VLPs at 10 µg/mL for 24 h, and the percentages of cells positive for IL-2 and IL-12, as markers of activation, were determined by flow cytometry (Fig. 2A and B). Compared with mock-treated cells, treatment of cells with WT VLPs resulted in an increase in percentages of IL-2^+^ cells from 0.06% to 1.81% (Fig. 2A) and IL-12^+^ cells from 3.16% to 7.33% (Fig. 2B), supporting the previous observations (Fig. 1C to E). When cells were exposed to mut 5 (which corresponds to GP K588A) VLPs, a reduction in the percentages of IL-2^+^ cells (1.04%) (Fig. 2A) and IL-12^+^ cells (3.76%) (Fig. 2B) was observed when compared with WT VLP. In contrast, mut 14 (which corresponds to GP W597A) induced the highest percentages of IL-2^+^ cells (5.67%) (Fig. 2A) and IL-12^+^ cells (14.56%) (Fig. 2B). In parallel, we evaluated the ISD activity on the expression of known anti-inflammatory mediators IL-10 (Fig. 2C) and cyclic AMP (cAMP) (Fig. 2D). Although the anti-inflammatory activity of IL-10 is well known (27), cAMP was demonstrated to be implicated for the immunosuppressive activity of ISD in retroviruses (21, 28, 29). cAMP is also known to inhibit cell activation and pro-inflammatory cytokine production (30–32). As such, we tested the amounts of IL-10^+^ and cAMP^+^ cells cultured for the ISD mutants. Strikingly, the mut 5 VLPs demonstrated the highest percentages of IL-10^+^ (7.28%) compared with WT VLPs (5.71%) (Fig. 2C) and cAMP levels (60% increase over WT) (Fig. 2D). In contrast, mut 14 VLPs demonstrated the lowest percentage (3.94%) of IL-10^+^ cells compared with WT and a 35% reduction of cAMP levels (Fig. 2C and D), consistent with an opposite trend in the cytokine responses. To determine the effects of the ISD amino acids 5 and 14 in the context of live EBOV infection, we introduced each mutation in the full-length clone encoding EBOV expressing GFP (28) (EBOV-GFP) and attempted to recover the associated virus, along with the control clone without the mutation, as previously described (29). Two attempts resulted in no viable virus recovered from the mutated constructs, whereas the WT control virus without a mutation was easily recovered. These data suggest that K588 and W597 in the ISD are critical for the viability of EBOV. Overall, our data further support the identification of K588 and W597 within the ISD as the residues responsible for its immunomodulating properties.

### The ISD Lys-5 and Trp-14 residues have opposite effects on the activation of PBMCs

**Fig 3:**
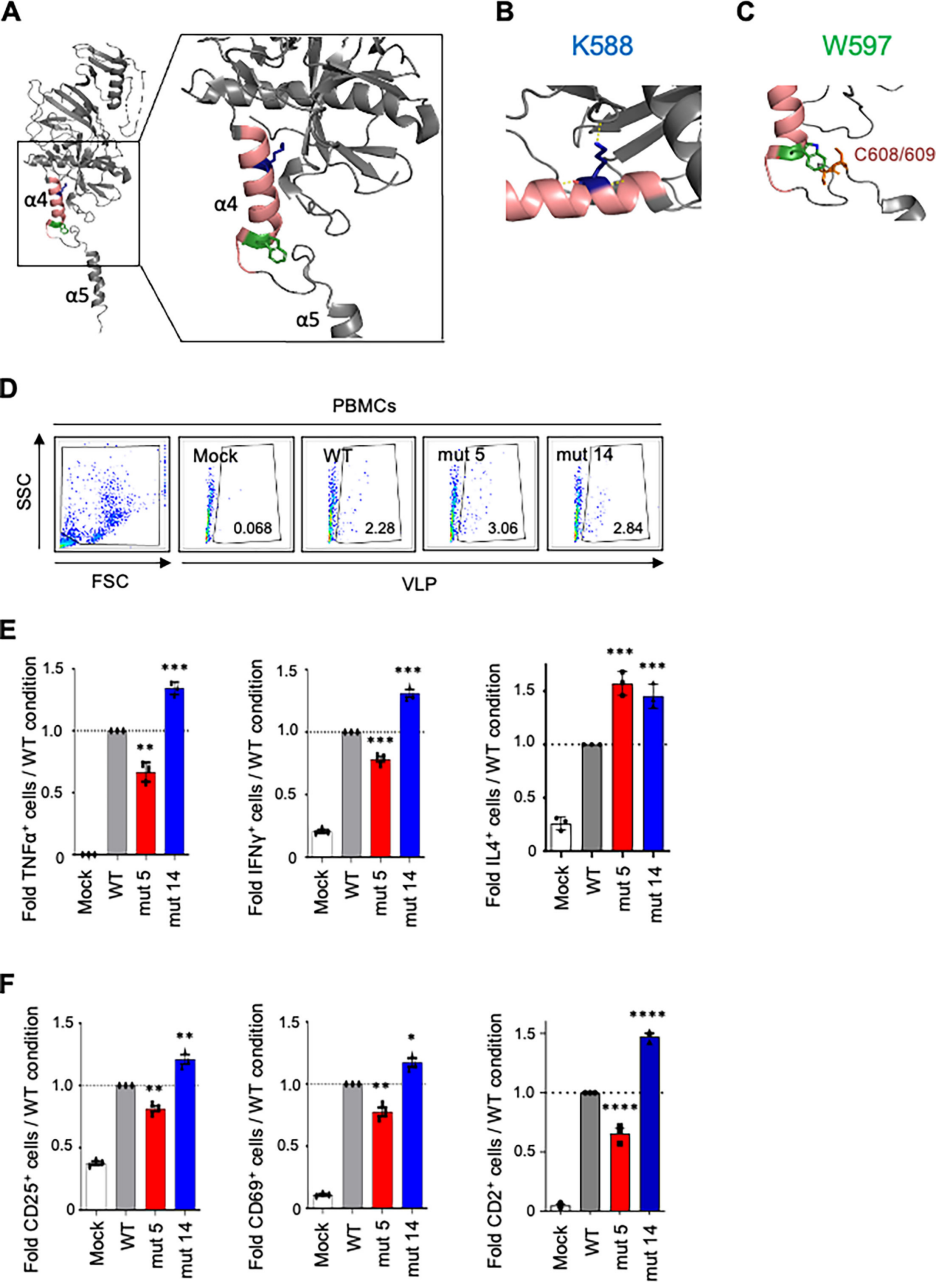
The N-K588 and W597C-terminal amino acids of EBOV GP ISD are responsible for the opposite effects on activation of T-cells in PBMCs. (**A**) 3D structure analysis of EBOV GP protein. The enlarged area shows the ISD in which the α-helix is marked with magenta, wherein mut 5 (K to A) is shown as a blue stick, and mut 14 (W to A) is shown as a green sheet. (**B, C**) Enlarged representation of K588 (**B**) and W597 (**C**). (**D**) Evaluation of WT or mut 5 and mut 14 VLP binding to PBMCs through flow cytometry analysis. (**E, F**) Flow cytometry analysis for TNFα, IFNγ, and IL4 (**E**) and CD25, CD69, and CD2 (**F**) in PBMCs mock-treated or cultured with WT, mut 5, or mut 14 VLP for 24 h. Data are represented as mean ± SEM from three independent experiments, with each donor sample analyzed in technical triplicate. All samples were analyzed using one-way ANOVA followed by a Tukey’s multiple comparison test: *P < 0.05, **P < 0.01; ***P < 0.001, ****P < 0.0001; ns, non-significant.

Computational analysis of the 3D structure of GP in relation to immunosuppressive domain (ISD) with greater detail for K588 and W597 is shown in Fig. 3A through C. The sequences of GP and the ISD mutants were analyzed using PyMOL software, an open-source molecular visualization system, for amino acid interactions. In the α-helix of ISD, the hydrogen bonding of K (lysine) contributes to the ability of α-helix 4 to interact with the N-terminal half of the protein, whereas W (tryptophan) maintains interaction with α-helix 5. The mutation to either K or W could maintain an intact interaction with the respective regions. As noted above, EBOV GP mediates viral attachment and entry into the host cells. We therefore tested whether the mutations have any impact on attachment, which could affect the cytokine response. First, we tested cell binding using human PBMCs. For that, human PBMCs were isolated and cultured with WT or mut 5 or mut 14 EBOV VLPs for 2 h at 4°C. Then, the cells were harvested, and percentages of cells with bound WT, mut 5, and mut 14 EBOV VLPs were quantified by flow cytometry. Equivalent levels of binding of WT and mutated VLPs were detected (Fig. 3D). Moreover, these data were confirmed when our analysis focused specifically on isolated B- and T-cell populations (Fig. S2A and B). These data suggest that mutations K588A and W597A do not have any strong effects on VLP-cell interactions. Next, we tested how the mutations impact the expression of inflammatory cytokines or activation markers, focusing on the T-cell population. Human PBMCs were mock-treated or cultured with WT or mut 5 or mut 14 VLPs for 48 h, and expressions of TNFα, IFNγ, IL-4 (Fig. 3E), CD25, CD69, and CD2 (Fig. 3F) were evaluated by flow cytometry. Consistent with the previous data, WT VLPs induced an increase in all these molecules compared with mock-treated cells. Mut 5 VLPs induced a reduced level of TNFα^+^ (33.4% compared with WT VLPs), IFNγ^+^ (21.7%), CD25^+^ (19.7%), CD69^+^ (22.4%), and CD2^+^ (34.4%) cells compared with WT VLPs. In contrast, mut 14 VLPs induced increased levels of TNFα^+^ (34.3%), IFNγ^+^ (31%), CD25^+^ (21%), CD69^+^ (17.3%), and CD2^+^ cells (47.3%) compared with WT VLPs. Interestingly, in comparison to WT VLPs, both mut 5 and mut 14 VLPs induced an increase in levels of IL-4 (57.3% and 45.3%, respectively), which is a pivotal cytokine involved in multiple immune mechanisms in either pro- or anti-inflammatory environments (30, 31). As the mutations did not affect the binding of VLPs to cells (Fig. 3D), the observed changes in the expression of cytokines and activation markers associated with the mutations are due to the cell-intrinsic processes in response to exposure to VLPs.

### The ISD Trp-14 residue reduces GP-induced inflammation mediated by the ISD Lys-5 residue

**Fig 4:**
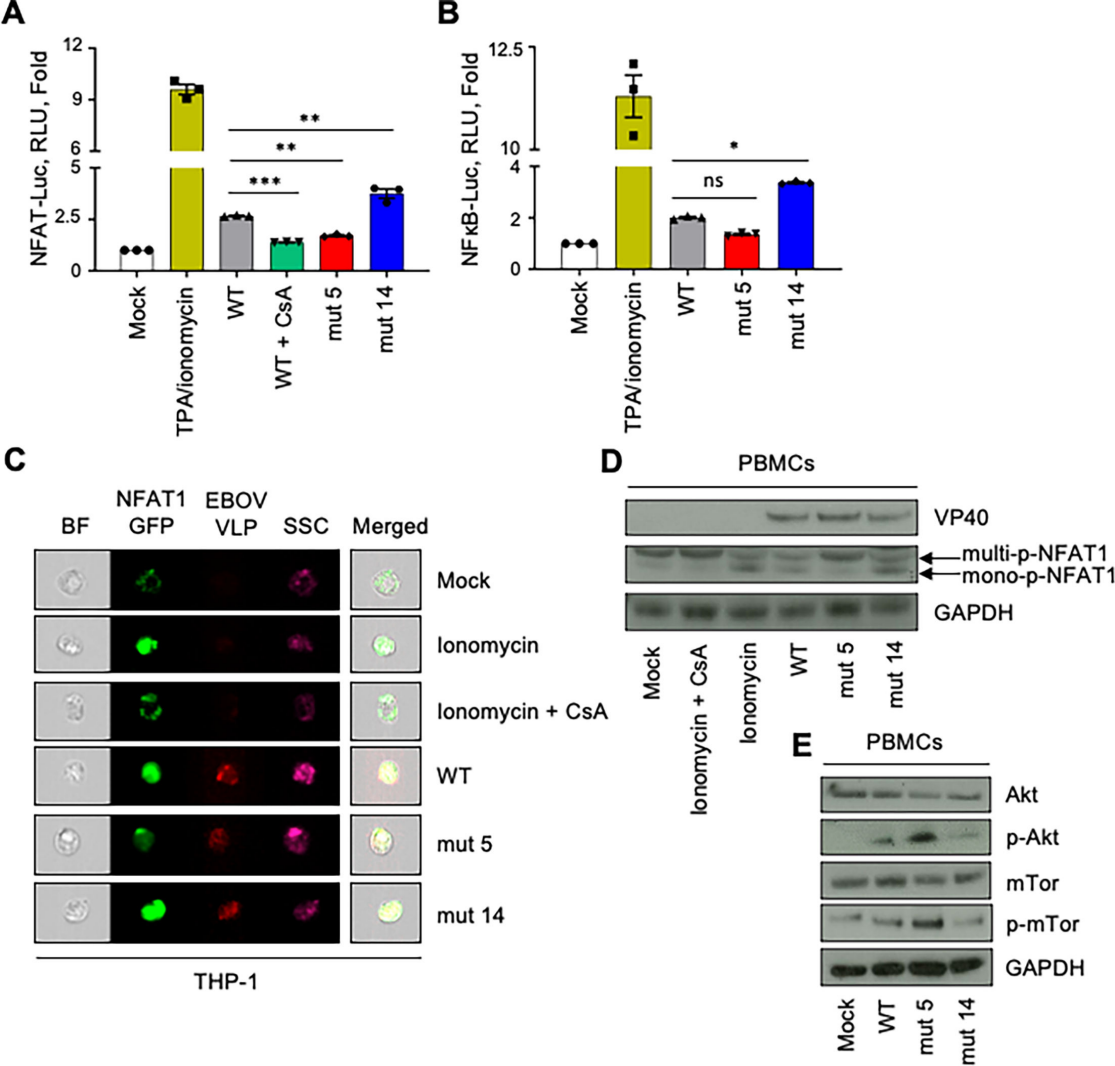
Trp-14 in the C-terminus of EBOV GP ISD mediates anti-inflammatory properties. (**A, B**) Transcriptional activity of NFAT1 and NF-kB in 293T cells stimulated with WT or ISD-mutated VLPs. 293T cells were transfected with NFAT-Luc (**A**) or NFκB-Luc plasmids (**B**). Then, the cells were stimulated or mock-stimulated with TPA + ionomycin, treated, or mock-treated with CsA for 1 h and treated or mock-treated with WT, mut 5, or mut 14 VLP. One-way ANOVA followed by a Tukey’s multiple comparison test: *P < 0.05; **P < 0.01; ***P < 0.001; ns, non-significant. (**C**) Imaging flow cytometry analysis of NFAT1-GFP (green) activation in THP-1 cells transfected with NFAT1-GFP and mock-treated, or treated by ionomycin alone or combined with CsA, or cultured with WT, mut 5, and mut 14 VLP (red). (**D**) Western blot analysis of NFAT1 phosphorylation profile (stained with anti-NFAT1) on PBMCs mock-treated or treated with ionomycin alone or combined with cyclosporine A (CsA), or cultured with WT, mut 5, and mut 14 VLP (stained with anti-VP40 antibodies). GAPDH was used as an internal control. (**E**) Western blot analysis of Akt phosphorylation profile (stained with anti-Akt and anti-phospho-Akt) and mTor phosphorylation profile (stained with anti-Akt and anti-phospho-Akt) on PBMCs mock-treated or treated by ionomycin alone or in combination with CsA (1 µM), or cultured with WT, mut 5, and mut 14 VLP (stained with anti-VP40 antibodies. GAPDH was used as an internal control.

It is well established that viral ISDs inhibit inflammatory responses (18–22, 25, 32). Consistent with our previous observations, the C-terminal mut 14 appeared to have a pro-inflammatory effect, suggesting an anti-inflammatory effect of the Trp-14 residue. Unexpectedly, our analysis demonstrated that the mut 5 ISD has an anti-inflammatory effect compared with WT, suggesting a pro-inflammatory effect of the specific Lys-5 residue in the WT sequence, contrasting the expected role of ISD. We therefore tested the effects of both mutations on the activity of the transcription factors involved in inflammatory responses (Fig. 4). Indeed, NF-κB and NFAT1 are major transcription factors that play important roles in the induction of cytokine gene expressions, cell activation, and the implementation of an inflammatory environment (33–38). First, luciferase assays demonstrated modulations in the profiles of NFAT1 expression (Fig. 4A) similar to the ones elicited for cytokines and activation markers previously tested in PBMCs (Fig. 3E and F). Specifically, mut 5 VLPs had reduced the activity of NFAT1 to levels comparable with the effect of cyclosporine A (CsA) and reduced the activity of NF-κB. In contrast, mut 14 VLPs demonstrated elevated activities compared with WT VLP (Fig. 4A and B).

Next, imaging flow cytometry experiments were performed to analyze the nuclear localization of NFAT1-GFP in monocytic THP-1 cells. Unlike mock-treated cells, cells cultured with WT VLP displayed the presence of NFAT1-GFP in the nucleus (Fig. 4C), thus representing its active profile. Cells stimulated with ionomycin (positive control) or mut 14 VLPs demonstrated increased nuclear localization of NFAT1-GFP compared with WT VLP. In contrast, cells exposed to mut 5 VLPs demonstrated reduced nuclear localization of NFAT1-GFP (Fig. 4C). These data paralleled the effects of the mutations on cytokine expression (Fig. 1 and 2). We hypothesized that the pro-inflammatory effect of the ISD N-terminus is associated with its induction of the active mono-phosphorylated form of NFAT1 (39). To test the hypothesis, human PBMCs were cultured with WT, mut 5, or mut 14 VLPs for 24 h, lysed, and analyzed by western blotting for the transcriptionally active mono-phosphorylated NFAT1 and its inactive multi-phosphorylated form. Ionomycin alone, which is a potent stimulator of cell proliferation, induced mono-phosphorylated NFAT1, whereas CsA completely inhibited its activity. Importantly, WT VLPs elevated the levels of mono-phosphorylated NFAT1 in PBMCs, concomitantly with previous observations. Moreover, PBMCs cultured with mut 5 VLPs did not elicit any significant activation of NFAT1 as seen by high levels of multi-phosphorylated form, but cells cultured with mut 14 VLPs displayed high levels of mono-phosphorylated NFAT1 equivalent to that induced by ionomycin (Fig. 4D).

**Fig 5:**
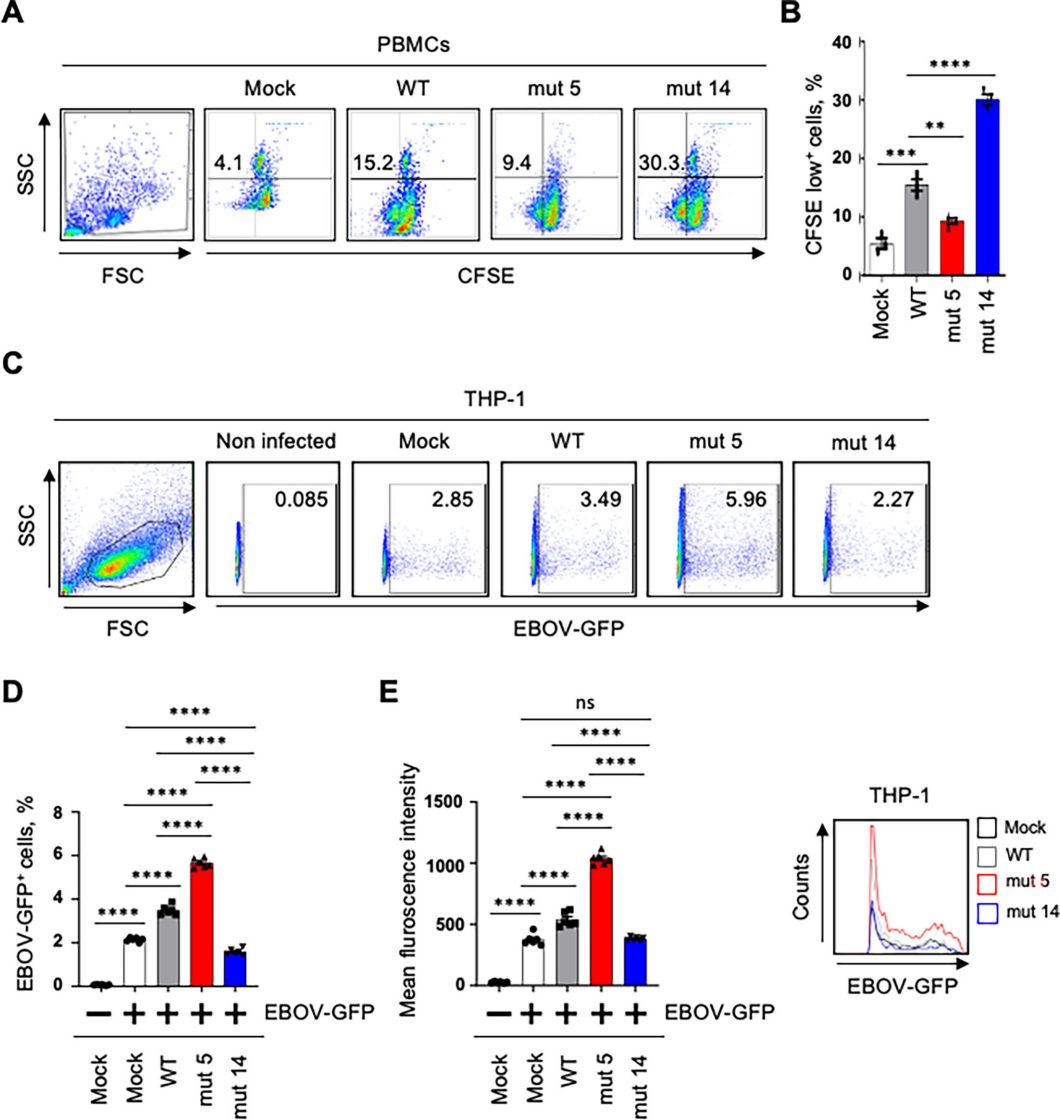
EBOV GP ISD contributes to cell proliferation and EBOV replication. (**A, B**). Flow cytometry analysis of PBMCs proliferation: primary data (**A**) and percentages of CFSE low^+^ cells (**B**). (**C–E**) Flow cytometry analysis of EBOV infection in VLP-treated THP-1 cells: primary data (**C**), percentages of GFP^+^ (infected) cells (**D**), and mean fluorescence intensities (left) and representative primary data (right) (**E**). Data are represented as mean ± SEM from three independent experiments with different donors, with each donor sample analyzed in technical triplicates. All samples were analyzed using one-way ANOVA, followed by a Tukey’s multiple comparison test: **P < 0.01; ***P < 0.001; ****P < 0.0001; ns, non-significant.

To further investigate the properties of EBOV GP ISD on anti-inflammatory-related signaling, we tested the activation of both the mammalian target of rapamycin (mTOR) protein and protein kinase B (Akt) (40). Human PBMCs were incubated with WT, mut 5, or mut 14 VLPs for 24 h, lysed, and analyzed by western blotting for the expression of active p-Akt and p-mTOR. WT VLPs cultured with PBMCs increased the levels of p-mTOR and p-Akt compared with the mock-treated condition (Fig. 4E). Contrary to what was described when investigating pro-inflammatory factors NFAT1 and NF-κB (Fig. 4A, B, and C), the levels of anti-inflammatory factors p-Akt and p-mTOR were increased in the presence of mut 5 VLPs while decreased in the presence of mut 14 VLPs when compared with that of WT VLPs (Fig. 5E). Overall, these data suggest that the N-terminal part of EBOV GP ISD induces pro-inflammatory pathways, whereas the C-terminal part exerts an anti-inflammatory signaling.

### EBOV GP ISD N- and C-termini have opposite effects on cell proliferation and viral replication

Next, we tested whether the mutations in ISD affect cell proliferation. To measure the effects of the mutations on cell division, human PBMCs were pre-labeled with carboxyfluorescein succinimidyl ester (CFSE), incubated with the different VLP constructs for 48 h, and the dilution of CFSE as a measure of cell proliferation was quantified by flow cytometry. Cells treated with mut 5 VLP demonstrated a reduction in cell proliferation, compared with WT VLPs, whereas mut 14 VLP-treated cells elicited an increase in proliferation (Fig. 5A and B). The immunosuppressive effect of the ISD C-terminus, based on the mut 14 response, is consistent with the suppression of cell proliferation previously described for the EBOV GP ISD-specific 17-mer peptide (27). These data suggest that the ISD C-terminus, which has an immunosuppressive effect, also inhibits cell proliferation.

Our previous studies have demonstrated that the direct binding of EBOV GP to TLR4 on monocytes promoted their differentiation, thus leading to their increased susceptibility to EBOV infection (13, 41). Therefore, we evaluated the effects of the mutations on viral replication in THP-1 monocytic cells. THP-1 cells were incubated with WT or the mutated VLPs for 24 h and infected with EBOV-GFP for 48 h. Next, viral replication was assessed by flow cytometry by quantitation of the percentages of infected (GFP+) cells and the mean fluorescent intensity (MFI) (Fig. 5C to E). Pre-treatment of THP-1 cells with WT VLPs favored an increased EBOV infection compared with mock-treated cells based on both percentages of GFP^+^ cells and the MFI, thus confirming our previous results (13) (Fig. 5C and D). Furthermore, pre-culture with mut 5 VLP increased the infection compared with WT VLP, whereas pre-treatment with mut 14 VLP reduced it (Fig. 5C to E). These data show opposite effects of the N- and C-termini on the susceptibility of THP-1 cells to EBOV infection, which are consistent with the results on cytokine expression (Fig. 1D and E, 2, and 3) and the activation of different signaling pathways (Fig. 1C and 4). Taken together, these results demonstrate that the ISD N-terminus induces a pro-inflammatory cellular environment, promotes cell proliferation, but reduces EBOV infection. In contrast, the ISD C-terminus promotes an anti-inflammatory environment, limits cell proliferation, but increases EBOV infection.

### EBOV GP increases the adhesion of monocytes to endothelial cells, and the effect is inhibited by the ISD C-terminus

**Fig 6:**
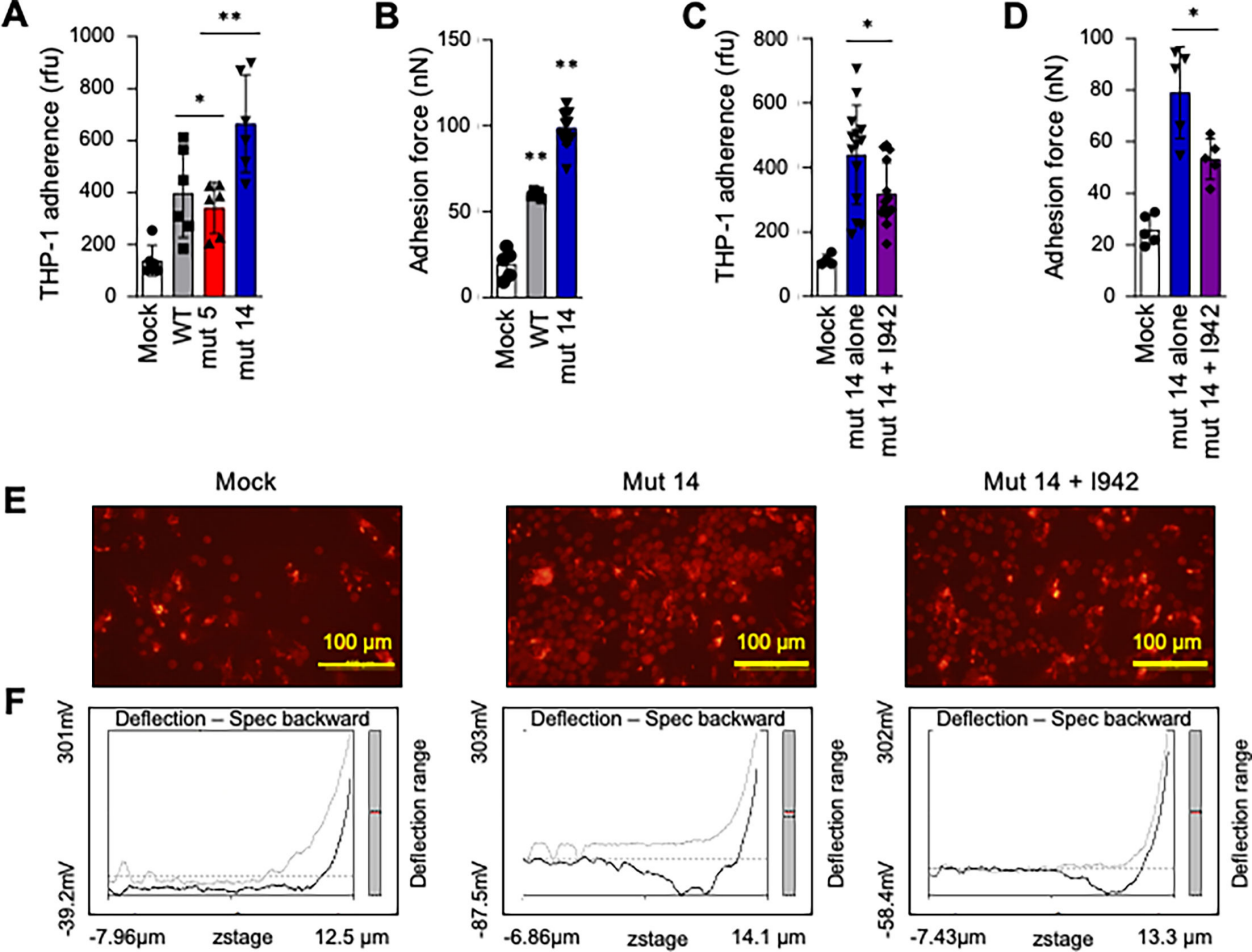
EBOV GP ISD Trp-14 inhibits monocyte adhesion to microvascular endothelial cells. (**A**) Fluorescent cell tracer-based adhesion assay with THP-1 and brain microvascular endothelial cell (BMEC): calcein-AM-labeled THP-1 monocytes (highlighted in red in representative images in panel E) were added to BMEC monolayers. The co-cultures were exposed to different VLPs for 72 h and fixed for image analysis to calculate rfu. The data represent the relative fluorescence unit (rfu) of adhered THP-1 cells on a monolayer of BMEC. *N* = 9. (**B**) Fluidic AFM studies measuring the adhesion force between a living THP-1 cell and a single living human BMEC. THP-1 cells and BMECs were separately exposed to WT-VLPs (*n* = 5) or Mut-14 -VLPs (*n* = 18) for 12 h. After that, VLP-treated THP-1 cells were added onto the BMEC monolayers. The cell mixtures in fresh normal endothelial culture media were loaded onto the fluidic AFM system to measure the vertical binding force between a single living THPI and a single living BMEC. The unbinding effort was assessed by measuring the work done (pJ), which was calculated by integrating the area under the force-distance (F–D) curve (representative curves in panel **E**). (**C**) Pharmacological activation of EPAC-1 attenuates mut-14-triggered monocyte-microvascular endothelial cell binding. Calcein-AM-labeled THP-1 cells and BMEC monolayer were co-cultured and exposed to different VLPs for 6 h. The cells were treated with EPAC-1-specific activator ESA I942 for 66 h. Cells were fixed, and rfu was calculated. *N* = 17. (**D**) Fluidic AFM studies measuring the adhesion force in THP-1 and BMEC. THP-1 and BMEC were separately exposed to 0.3 µg/mL mut-14 VLP for 6 h and treated with ESA I942 for an additional 6 h. Treated THP-1 cells were added onto the BMEC. The cell mixture was loaded onto the fluidic AFM system to measure the vertical binding force between a single living THP-1 and a single living BMEC using a micropipette. Mock-treated cells served as the control. The unbinding effort was assessed by measuring the work done (in pJ), which was calculated by integrating the area under the force-distance (F-D) curve. *N* = 5. (**E**) Representative images of fluorescent cell tracer-based adhesion for panel D. Monocyte adhesion visualized by fluorescent microscopy. After co-cultures for a designed time, non-adherent monocytes were washed off, and adherent monocytes were fixed. Calcein-AM-labeled THP-1 monocytes are visualized in red. (**F**) Representative fluidic AFM force-distance curves (F-D curves) for measuring vertical binding forces between single THP-1 cells and BMEC in different groups in panel. **D**. Each panel represents an F-D curve obtained from a single set of THP1-BMEC interactions, measured during the unbinding of the cell-probe from the BMEC surface by retracting the Fluidic AFM cantilever. Data represent mean ± SD. *P < 0.05; **P < 0.01, one-way ANOVA.

As demonstrated previously, mut 14 VLPs enhanced the expression of pro-inflammatory cytokines and decreased the cAMP levels compared with WT VLPs, thus suggesting EBOV GP ISD Trp-14 anti-inflammatory property (Fig. 2). The anti-inflammatory properties of cAMP are mediated by exchange protein directly activated by cAMP type 1 (EPAC-1), which is an intracellular cAMP receptor (42–44). The cAMP-EPAC1 axis suppresses the inflammatory process by stabilizing microvascular endothelial cells in inflammation (45). The adhesion of leukocytes to the microvascular endothelium, prior to their infiltration into the perivascular space, is a key characteristic of focal inflammation (46). In the bloodstream, leukocytes must strongly attach to the endothelial surface to overcome detachment by shear stress from blood flow. Therefore, leukocyte adhesion can be investigated by measuring real-time nanoscale binding force between living leukocytes and microvascular endothelial cells. Fluidic AFM technology is particularly well suited to achieve this goal (47). Using a conventional monocyte adhesion assay (48) and fluidic AFM, we tested whether the ISD and its mutations affect the monocyte-endothelial cell adhesion as part of the inflammatory process. For that, THP-1 monocytes were labeled with the fluorescent dye Calcein-AM and added atop a human brain microvascular endothelial cell (HBMVEC) monolayer, and the co-cultures were exposed to WT or mutant VLPs (0.3 µg/mL) followed by incubation for 72 h. Next, the cells were washed with phosphate-buffered saline (PBS), and the relative fluorescence intensities for the number of Calcein-AM-labeled THP-1 cells adhered to the monolayer of HBMVEC were scored using relative fluorescence units (rfu) (49). We found that WT VLPs increased THP-1 adherence to HBMVEC cells compared to mock-treated cells, and mut 5 demonstrated only a marginal reduction of the adherence. In contrast, mut 14 demonstrated further increased adherence (Fig. 6A). Therefore, mut 14 was further characterized by measuring the adhesion force between single-live THP-1 and single-live HBMVEC cells by fluidic atomic force microscopy (fluidic AFM). Again, we observed increased adhesion forces for mut 14, compared with WT VLPs (Fig. 6B). Thus, the anti-inflammatory effect of Trp-14 in WT ISD is accompanied by reduced monocyte adhesion.

We hypothesized that the higher monocyte-endothelial cell adhesion by mut 14 can be associated with a reduction of cAMP, resulting in a decrease of EPAC-1 levels (45). To test this hypothesis, we incubated THP-1-HBMVEC cocultures with mut 14 VLP for 6 h and then treated them with ESA I942, a specific activator of EPAC-1 expression, to measure cell-cell adhesion. As expected, the activation of EPAC-1 significantly reduced monocyte-endothelial cell adhesion as measured by lower rfu of THP-1 cells (Fig. 6C, E, and F). Similarly, the fluidic AFM analysis confirmed that the activation of EPAC-1 by ESA I942 reduced the adhesion force between THP-1 and HBMVEC (Fig. 6D). Thus, mut 5 demonstrated an intact cAMP-EPAC-1 axis, which protects barrier function, contributing to reduced inflammatory response. In contrast, mut 14 demonstrated a compromised cAMP-EPAC-1 axis contributing to the pro-inflammatory response. Overall, these data demonstrate that ISD facilitates the adhesion of monocytes to endothelial cells mediated by EPAC-1, presumably as a consequence of its overall anti-inflammatory effect mediated by its C-terminus.

### The N- and C-termini of shed GP ISD trigger expression of pro- and anti-inflammatory cytokines, respectively

**Fig 7:**
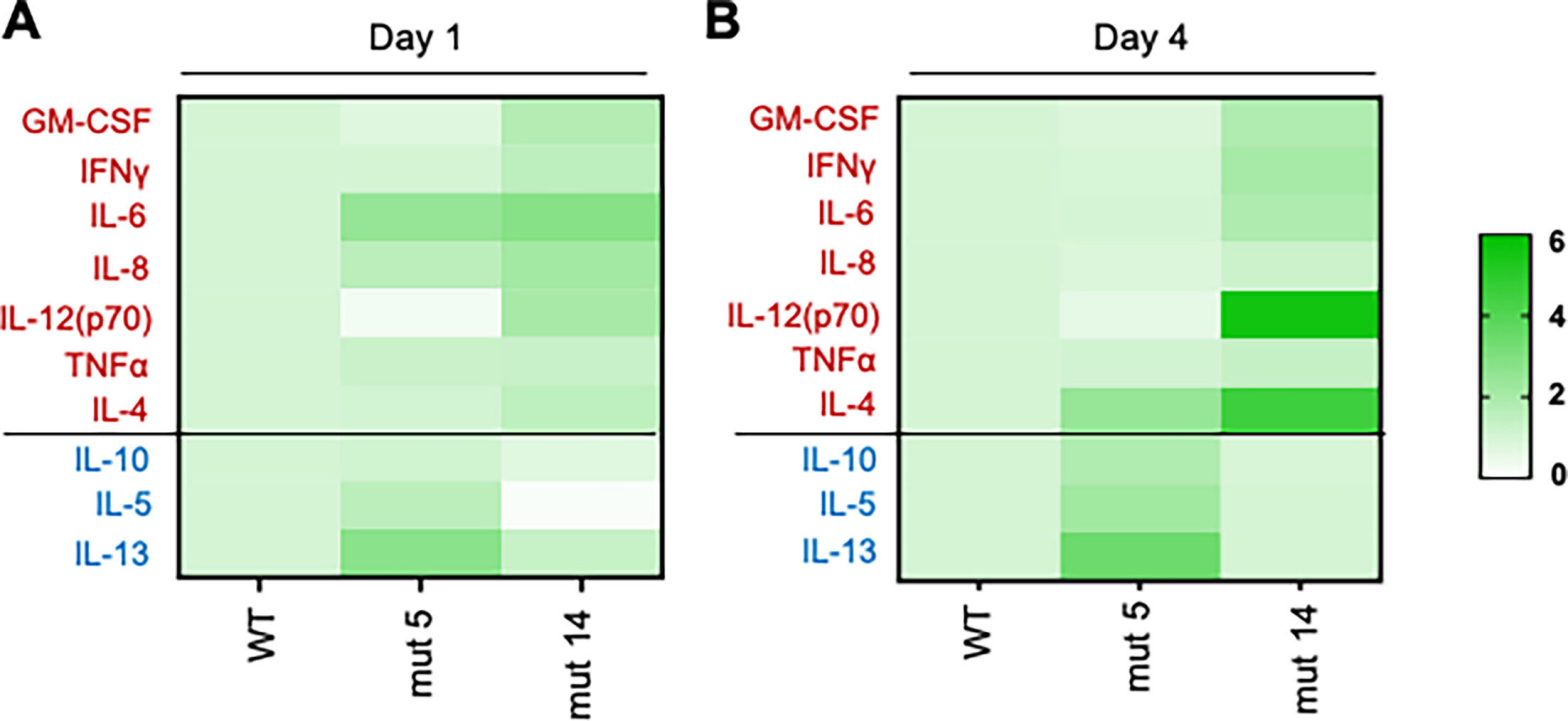
N- and C-termini of EBOV shed GP ISD differentially trigger the expression of pro- and anti-inflammatory cytokines. Multiplex assay quantifying pro-inflammatory cytokines (indicated in dark red) and anti-inflammatory cytokines (indicated in blue). PBMCs were mock-treated or cultured with WT, mut 5, and mut 14 EBOV shed GP for 24 h (**A**) or 96 h (**B**). The values were normalized on WT shed GP cultures. Representative data from three independent experiments with PBMCs from individual donors.

We have characterized the effects of ISD on EBOV GP exposed on the surface of VLPs, thus mimicking the effects of GP on the surface of EBOV particles. However, an alternative form of GP is released into the extracellular medium from infected cells (11). Specifically, during EBOV infection, the GP ectodomain is cleaved off by TACE, shed extensively, and triggers immune cell activation and cell death (13, 50). We hypothesized that ISD in shed GP contributes to immune modulation. To test this hypothesis and investigate the role of the N- and C-termini of the ISD in the context of shed GP alone without any other viral factor, we generated WT, mut 5, and mut 14 shed GP proteins as described previously (13) and incubated them in PBMC cultures for 24 h and 96 h. The supernatants were harvested and analyzed by bead-based multiplex assay to measure the levels of secreted pro- and anti-inflammatory cytokines. Globally, WT, mut 5, and mut 14 shed GPs increased both pro- and anti-inflammatory cytokine levels in comparison to mock-treated PBMCs on both day 1 and day 4 (Fig. S3A and B), thus confirming the previous results obtained with VLPs. A more detailed analysis demonstrated that in comparison to WT shed GP, mut 5 shed GP increased mostly anti-inflammatory cytokines, specifically IL-10, IL-5, IL-13, and IL-4, particularly on day 4 (Fig. 7). In contrast, mut 14 shed GP mostly increased pro-inflammatory cytokines, including GM-CSF, IFNγ, IL-8, IL-12 (p70), IL-6, TNFα, and IL-4. Induction of IL-4 by both mutants is consistent with the observations showing induction of IL-4 by both mut 5 and mut 14 VLPs (Fig. 3E). Overall, the pattern of cytokine expression by shed GP mutants was similar to that of the VLP mutants (Fig. 1C through E left panels, Fig. 2 and 3E). Altogether, these data demonstrate that the ISD present in shed GP is capable of modulating the immune response with its N- and C-termini having pro- and anti-inflammatory properties, respectively.

### EBOV shed GP is internalized through interaction with TLR4 and modulates its downstream signaling pathway

**Fig 8:**
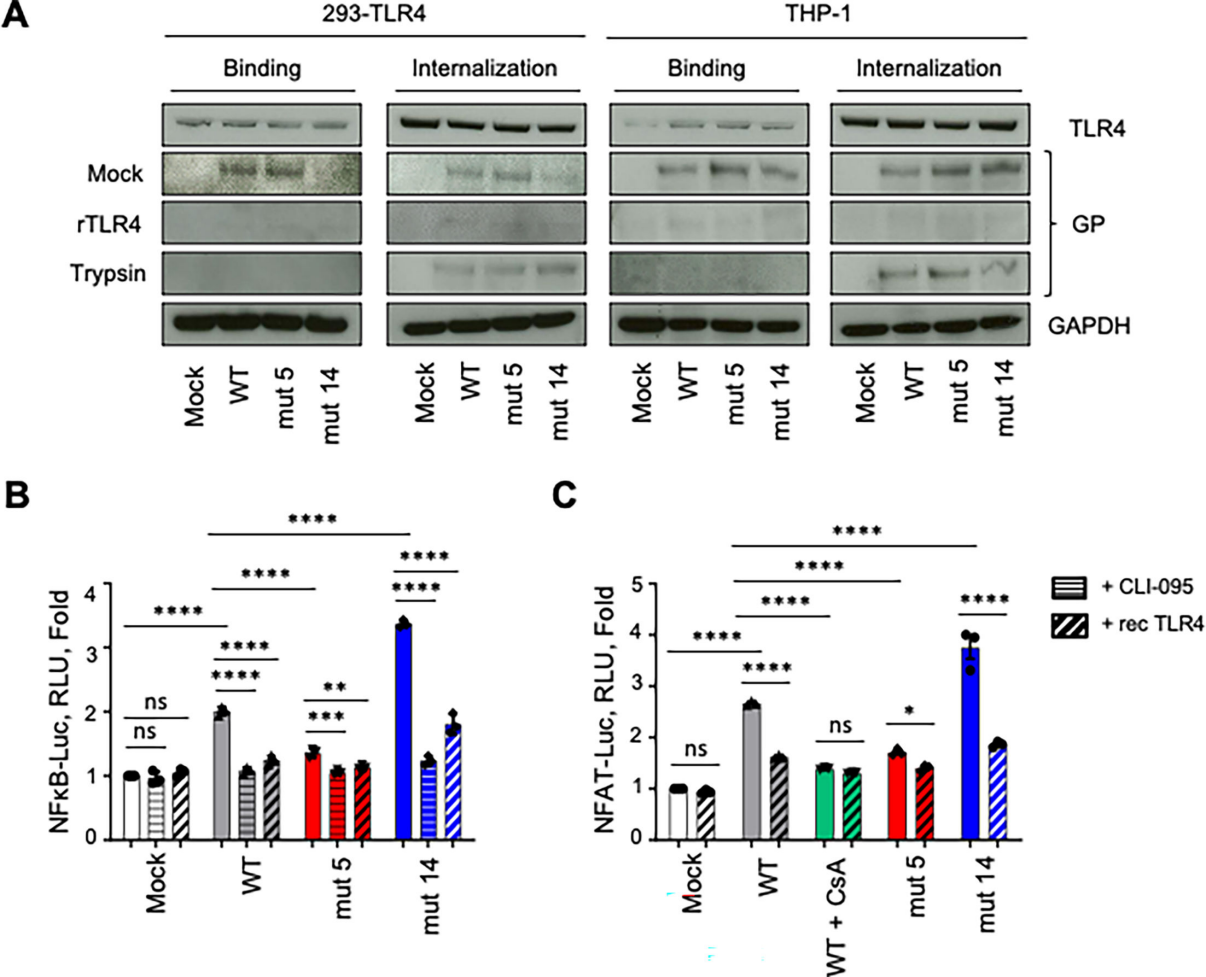
TLR4 is involved in the capture of shed EBOV GP. (**A**) Binding and internalization assays on 293 cells stably expressing TLR4 (293-TLR4) and THP-1 cells were analyzed by western blotting. Cells were treated or mock-treated with recombinant TLR4 (rTLR4) and then cultured with medium or WT, mut 5, and mut 14 EBOV shed GP. Cells were then treated with trypsin to evaluate internalization of shed GP. Cell pellets were immunostained for TLR4, EBOV GP, and GAPDH as an internal control. (**B, C**) Induction of NFκB and NFAT. 293-TLR4 cells were transfected with NFAT-Luc (**B**) or NFκB-Luc (**C**), treated with CLI-095 or rTLR4 with or without CsA, treated or mock-treated with WT, mut 5, or mut 14 EBOV shed GP, and subjected to luciferase assays. Two-way ANOVA followed by a Tukey’s multiple comparison test: *P < 0.05; **P < 0.01; ***P < 0.001; ****P < 0.0001; ns, not significant.

We previously reported that shed GP activates immune cells in a TLR4-dependent manner to promote cellular differentiation and viral replication (13). Here, we tested the role of shed GP ISD in the activation of cells through TLR4. First, to evaluate binding of each shed GP version (Fig. 8A, 1st and 3rd column), HEK 293 cells stably expressing TLR4 (293-TLR4), or THP-1 cells were mock-treated or treated with recombinant TLR4 (rTLR4) and exposed to WT or both mutated shed GPs for 2 h on ice to inhibit internalization. Then, to further investigate internalization (Fig. 8A, 2nd and 4th column), following the same procedure, the cells were washed and then either trypsinized for 10 min or mock-treated, and shifted to 37°C for 1 h to promote internalization. Thereafter, the cells were lysed, and the lysates were analyzed by western blotting with antibodies specific to GP or TLR-4. Exposure of cells to WT, mut 5, and mut 14 shed GP demonstrated no difference in cellular binding and internalization, as the GP signal remained unchanged (Fig. 8A, 2nd line), thus corroborating previous observations with VLPs (Fig. 3D; Fig. S2). However, pre-treatment with rTLR4 significantly reduced EBOV GP binding and its internalization (Fig. 8A, 3rd line). Treatment of cells with trypsin has stripped off cell membrane-bound GP, but not the internalized GP (Fig. 8A, 4th line). Together, these data demonstrate that TLR4 plays an important role in the internalization of shed GP and that mutations at positions 5 and 14 in the ISD do not have any impact either on its binding or its internalization.

Next, we determined the capacity of ISD mutations to modulate TLR4-related transcription factor activities involved in multiple cellular processes. 293-TLR4 cells were transfected with NF-κB-Luc and NFAT-Luc plasmids for 48 h and mock-treated or treated with the specific TLR4 signaling inhibitor CLI-095 or rTLR4 with or without CsA, a specific inhibitor of the NFAT signaling pathway. Then, the cells were cultured with WT, mut 5, or mut 14 EBOV shed GP for 24 h and subjected to luciferase assays. As demonstrated previously, WT EBOV shed GP activated both NF-κB and NFAT1 pathways in mock-treated cultures (Fig. 1C, 4A and B, and 8B and C). Shed GP mut 5 and mut 14 displayed reduction and increase in both NF-kB and NFAT activities, respectively (Fig. 8B and C). Moreover, to characterize the role of TLR4, we pre-treated cell cultures with the specific TLR4 signaling inhibitor CLI-095 or rTLR4, which significantly reduced the activities of both transcription factors perpetrated by the shed GPs (Fig. 8B and C). Together, these data demonstrate that following TLR4 engagement with EBOV shed GP, K588 promotes cellular activation and expression of pro-inflammatory cytokines via NF-kB and NFAT pathways, whereas W597 inhibits these effects.

## DISCUSSION

The ISD, a conserved region in the retroviral envelope glycoproteins, was shown to inhibit immune responses in multiple systems (18, 20–22). Likewise, EBOV and Marburg virus have a conserved ISD in their glycoprotein (17, 23, 24, 51–53). An *in vitro* study, which used a 17-residue peptide that mimics EBOV GP ISD, has demonstrated an immunosuppressive activity by inhibiting activation of CD4^+^ and CD8^+^ T cells (25). As already noted, a linear peptide treatment may not reproduce the biological effects of ISD in a fully conformational GP. As such, we first evaluated the effects of EBOV GP ISD on human PBMCs using EBOV VLPs consisting of the NP, VP40, and GP proteins. We demonstrated that VLPs decrease the transcriptional activity and cytokine expression in pre-stimulated PMBCs (Fig. 1C through E). To further investigate ISD properties, we generated 17 VLPs displaying single mutations through alanine or glycine substitutions in the full sequence of ISD. That strategy allowed us to identify two residues playing important roles in the modulation of inflammation (Fig. 2). Indeed, as mut 5 VLPs enhanced the induction of anti-inflammatory cytokines and reduced activation of T cells, the WT N-terminus sequence of ISD is likely to induce a pro-inflammatory response. In contrast, mut 14 VLPs elicited pro-inflammatory-associated cytokines and T-cell activation, thus suggesting that the WT C-terminus sequence of ISD is involved in its immunosuppressive properties. The pro-inflammatory effect of the ISD N-terminus is remarkable, as it is opposite to the expected immunosuppressive effects of the conserved ISD. These data demonstrate a balance and a possible dichotomy in the cellular response elicited by ISD.

**Fig 9:**
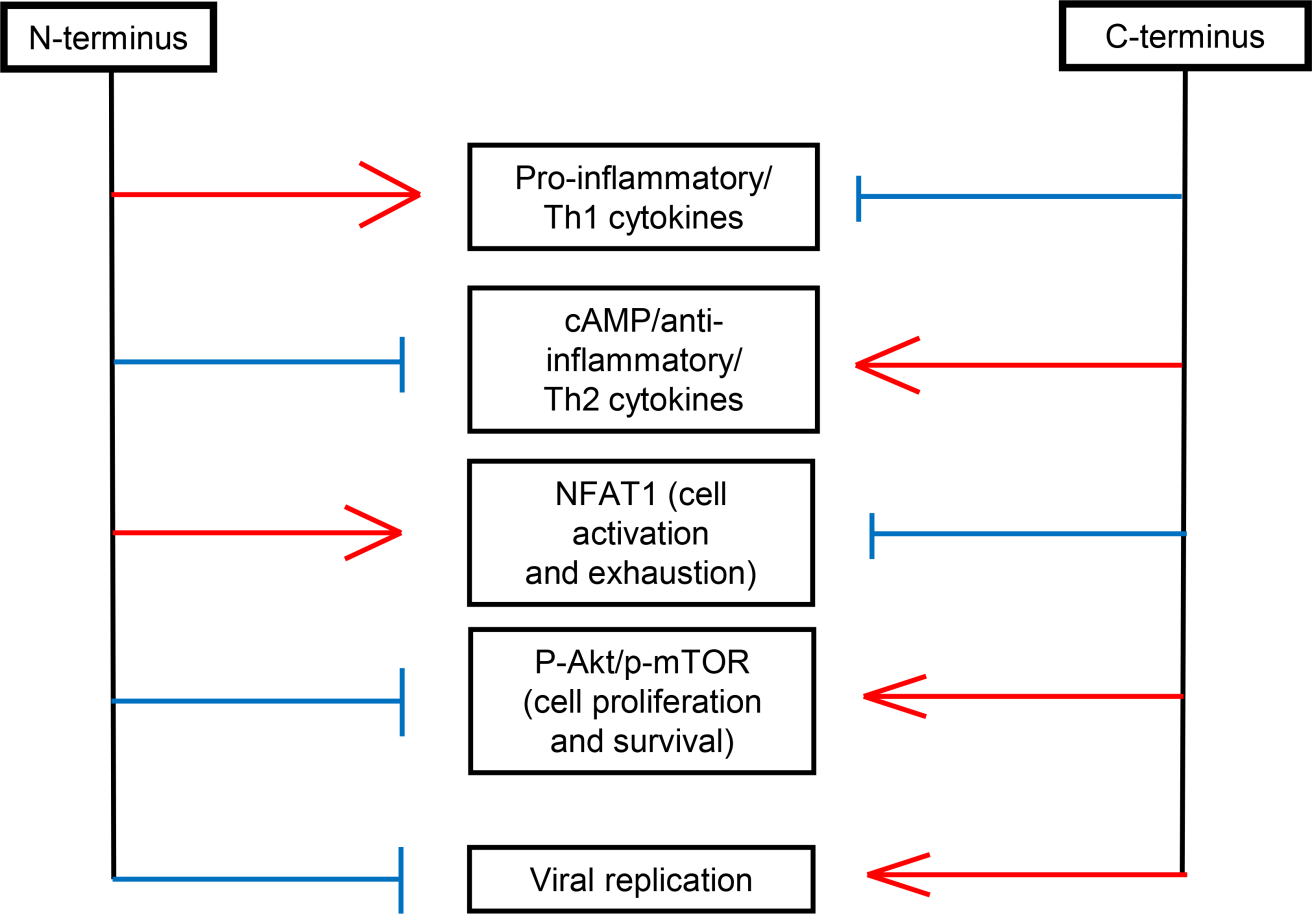
The N- and C-termini of EBOV GP ISD have opposite biological effects.

However, it remains unclear how the mutants with opposite effects impact the immune responses. We therefore tested the mutants for cell binding and entry, which did not show any significant difference between WT, mut 5, and mut 14 VLPs (Fig. 3D) and shed GPs (Fig. 9A). We then determined that following cell entry, the ISD mutants differently trigger signaling pathways, as mut 5 VLPs favored the activation of the anti-inflammatory Akt/mTor axis, whereas mut 14 VLPs triggered the pro-inflammatory NFAT and NF-κB pathways in comparison to WT VLPs. As our initial data demonstrate global differences in the induction of pro- and anti-inflammatory responses, we next tested the ability of the VLP mutants to alter cell proliferation and also viral replication. Consistent with cytokine profiles, cells cultured with mut 5 and mut 14 VLPs displayed reduced and increased cell proliferation, respectively. The effect of the mutations in the ISD on EBOV replication was opposite: increased in the presence of mut 5 and reduced in the presence of mut 14, potentially due to the characteristics of the immune environment induced by each mutant, thus hampering or enhancing the antiviral effects, respectively.

EBOV replicates in multiple cell types, resulting in a complex pathogenesis that includes an uncontrolled and unbalanced immune response leading to high fatality rates (3). Indeed, EBOV triggers hypercytokinemia, coagulopathy, vascular damage, and immune-mediated tissue pathologies (15, 54), contributing to the damage of vital organs (3). During the process of inflammation, both cytokine induction and breaks in cell-junction barriers facilitate the transmigration of pro-inflammatory cells through capillary endothelial cells (55). Vascular endothelial barrier function is maintained by several mechanisms, such as the one involving the cAMP-EPAC1 axis, which likely reduces inflammation, thus sustaining its shielding properties and minimizing transmigration and inflammation (45). We observed that mut 14 increased monocyte-endothelial cell adhesion with an elevated binding force when compared with the WT or mut 5 VLPs. As mut 14 also reduced cAMP, which acts through EPAC-1 (42, 56) to maintain vascular permeability, it is concluded that mut 14 could enhance vascular permeability and transmigration, as it has been demonstrated to promote pro-inflammatory response. These results suggest that the residue W597 reduces the inflammation, contributing to “immunosuppression.” The induction of cAMP by W597 can potentially shut down the proximal T-cell activation, which was previously reported as a negative regulator of T cells (57). On the other hand, the capacity of the residue K588 to keep cAMP at low levels can trigger inflammatory processes, including T-cell activation, cell-cell adhesion, and cellular transmigration.

During EBOV infection, shed GP, which is abundantly released from infected cells, activates bystander immune cells, resulting in excessive release of inflammatory cytokines, increased vascular permeability, and dysregulated inflammatory changes (13, 50). To test if this protein further amplifies the effects of GP anchored in viral particles, as demonstrated using VLPs, we generated EBOV shed GP WT, mut 5, and mut 14. Indeed, and similarly to the observations with EBOV VLPs, shed GP mutants did not show any difference in cellular binding and entry. Again, exposure of human PBMCs to the mutated shed GP resulted in differential cellular activation and cytokine response, as mut 5 promoted an anti-inflammatory response, whereas mut 14 enhanced pro-inflammatory response (Fig. S3; Fig. 7). Additional experiments demonstrated that shed GP mut 5 and mut 14 displayed reduction and increase in both NF-kB and NFAT activities, respectively (Fig. 8).

Overall, our data suggest that the ISD N-terminus plays a role in activating immune cells and pro-inflammatory response. In contrast, the C-terminus of ISD downregulates the pro-inflammatory response through the reduction of NF-kB and NFAT activities. Thus, the expected immunosuppressive and anti-inflammatory effects of the ISD are associated with its C-terminus, whereas the N-terminus has unexpected pro-inflammatory properties (Fig. 9). These data also show that EBOV GP increases the adhesion of monocytes to endothelial cells, and the effect is inhibited by the ISD C-terminus (Fig. 9). Moreover, the data show that the immunomodulating effects of ISD are mediated not only by the virus-associated GP but also shed GP, which is abundant in the medium. Although no molecular mechanism of action has ever been associated with EBOV GP ISD, evaluating its potential association with the receptors from the TYRO3 receptor tyrosine kinase family and its transduction signaling might be of interest. Indeed, TYRO3 receptors are responsible for activating the Akt/mTOR pathway, thus promoting an anti-inflammatory response (58, 59). Moreover, members of the Tyro3 receptor tyrosine kinase family are involved in cell entry of EBOV (60). Pathogenesis of EBOV disease is characterized by a paradoxical combination of hyperinflammation (61–68) and features of immunosuppression (29, 69–71), reviewed in ref (72), which could in part be affected by the complex effects of the ISD presented in this study. These data may be useful for the development of treatments for the disease caused by EBOV by targeting the ISD.

Our study has limitations. First, although VLPs reproduce the three-dimensional structure of EBOV particles, they cannot completely reproduce the complexity of the biological effects of ISD in the context of EBOV infection. Second, some of the effects found in this study, particularly the modulation of the adhesion of monocytes to endothelial cells, should be further investigated in the context of EBOV infection *in vivo*.

## MATERIALS AND METHODS

### Virus, VLPs, and shed GPs

The recombinant EBOV, strain Mayinga, expressing green fluorescent protein (EBOV-GFP) (28) was recovered from the cDNA and propagated by three passages in Vero E6 cell monolayers as previously described (29). The viral stocks were quantified by plaque titration in Vero E6 monolayers. All work with EBOV was performed in a biosafety level 4 (BSL-4) laboratory of the Galveston National Laboratory. Generation of EBOV VLPs was performed as described previously (13). The mutants coding for pcDNA3.1 mut 5 EBOV GP-eGFP and pcDNA3.1 mut 14 EBOV GP-eGFP were generated using the Q5 site-directed mutagenesis kit (New England Biolabs) according to the manufacturer’s procedure. EBOV VLPs were generated using the plasmids WT, mut 5, or mut 14 pcDNA3.1 EBOV GP-eGFP, VP40 pWRG7077:64759-2010-233-1-4_VP40_optVP40 provided by Dr. Sina Bavari (U.S. Army Medical Research Institute of Infectious Diseases), and EBOV NP pCEZ-NP provided by Dr. Yoshihiro Kawaoka (University of Wisconsin) following transfection in 293T cells using TransIT-LT1 Mirus reagent (Mirus Bio) for 72 h. Transfected cells were collected and centrifuged at 10,000 × *g* for 10 min to remove cell debris. Then, cell supernatants were purified by pelleting through a 20% (wt/vol) sucrose cushion at 100,000 × *g*, 4°C for 120 min using a Beckman ultracentrifuge. The pellets were resuspended in 20 mL of PBS and further purified by ultracentrifugation at 100,000 × *g*, 4°C for 60 min. In parallel, VLPs were also concentrated using the Lenti-X concentrator reagent (Takara) following the manufacturer’s protocol. Each suspension containing the VLPs was determined by western blotting. The total protein concentrations of the VLP or virus preparations were determined after lysis in Nonidet P-40 detergent by using a Pierce BCA assay (Thermo Fisher Scientific). Briefly, the protein concentration in samples was determined using BCA reagent and read at 562 nm. The BSA standards were used to determine the protein concentration in samples. Recombinant shed GPs were generated by transfection of WT, mut 5, or mut 14 pcDNA3.1 EBOV GP-eGFP plasmid in 293T cells using TransIT-LT1 Mirus reagent (Mirus Bio) for 72 h. Transfected cells were collected and centrifuged at 10,000 × *g* for 10 min to remove cell debris, and supernatants containing shed GP were concentrated using Centricon-Plus 70 centrifugal filter units (EMD Millipore) following the manufacturer’s recommendations. Concentration of shed GPs in each sample was determined using Pierce BCA Protein Assay (ThermoFisher Scientific). To ensure that we use equal concentrations of the shed GPs WT, mut 5, and mut 14, we performed western blot analysis using an anti-EBOV GP (Integrated BioTherapeutics, #0201-020), followed by densitometry analysis (ImageJ software) for normalization. In parallel, VLPs were characterized by western blot analysis using anti-EBOV VP40 (Integrated BioTherapeutics, #0301-010), anti-EBOV NP (Integrated BioTherapeutics, #0301-012), and anti-EBOV GP (Integrated BioTherapeutics, #0201-020).

### Cells

Human monocytic cells THP-1 obtained from the American Type Culture Collection (ATCC) and THP-1 Blue NF-κB cells (InvivoGen) were cultured in RPMI 1640 medium (Thermo Fisher Scientific) supplemented with 10% heat-inactivated fetal bovine serum (FBS) (GE Hyclone) and 1% HEPES (Corning). Vero-E6 and 293T cell lines (obtained from the ATCC) and HEK 293-TLR4 cell line (293/HTLR4a. InvivoGen) were cultured in Dulbecco’s modified Eagle’s medium, supplemented with 10% FBS (Thermo Fisher Scientific), 1% HEPES (Corning), 1% nonessential amino acids (Sigma-Aldrich), 1% sodium pyruvate (Sigma-Aldrich), and 2% penicillin-streptomycin mix (Thermo Fisher Scientific). Human brain microvascular endothelial cells (HBMVECs) (10HU-051, iXCells Biotechnologies) were grown in endothelial cell growth medium (Cell Applications) supplemented with 10% heat-inactivated FBS with humidity in 5% CO_2_ at 37°C. Cells were maintained in human endothelial cell growth medium during the experiments. The temperature controller (NanoSurf) kept the liquid environment at 37°C during all measurements.

### Isolation and culture of human PBMCs

Buffy coats were obtained from deidentified healthy adult donors according to a clinical protocol approved by the University of Texas Medical Branch at Galveston (UTMB) Institutional Review Board. PBMCs were isolated by Histopaque (Sigma-Aldrich) gradient as recommended by the manufacturer. Then, CD3^+^ T-cells and CD20^+^ B-cells were isolated from fresh PBMCs using a cell-specific negative selection enrichment kit (StemCell Technologies). Purity of the isolated lymphocytes typically ranged from 93% to 95% as determined by flow cytometry using a LSR Fortessa flow cytometer (BD Biosciences) at the UTMB Flow Cytometry Core Unit. Data were analyzed using FlowJo v8 (FlowJo, LLC).

### VLP treatment, cAMP level evaluation, and flow cytometry analysis of PBMCs

PBMCs were cultured at 10^6^ per milliliter and pulsed with EBOV VLPs at 10 µg/mL and maintained in RMPI‐1640 medium (Sigma-Aldrich) supplemented with 10% of heat‐inactivated FBS. As a control, cells were stimulated with TPA (Sigma-Aldrich) at 25 ng/mL and 0.5 µM of ionomycin (Sigma-Aldrich) or 10 µM Forskolin (Sigma-Aldrich). Two hours following pulsing, Brefeldin‐A (Sigma-Aldrich) was added at 10 µg/mL, and the cells were incubated for an additional 16 h at 37°C. Levels of cAMP in PBMCs were evaluated using a specific cyclic AMP ELISA kit (Cayman) following the manufacturer’s instructions. In parallel, PBMCs were washed twice with PBS by spinning at 250 × *g* for 5 min at 4°C and fixed with 0.1 mL 4% paraformaldehyde (Fisher Bioreagents). PBMCs were permeabilized with permeabilization buffer (eBiosciences), stained with antibodies specific for IL-2 (BD Biosciences, #341116), IL-10 (BD Biosciences, #554707), IL-12 (BD Biosciences, #554576), TNFα (BD Biosciences, #340534), IFNγ (BD Biosciences, #554702), or IL-4 (BD Biosciences, #554485), washed with PBS, and resuspended in 500 µL of PBS. To evaluate activation markers on T-lymphocytes, cells were surface-stained with antibodies specific for CD25 (BD Biosciences, #347643), CD69 (BD Biosciences, #340560), or CD2 (BD Biosciences, #555327) for 30 min and washed. Flow cytometry was performed using a LSR Fortessa flow cytometer (BD Biosciences) at the UTMB Flow Cytometry Core Unit, and data were analyzed using FlowJo.

### Cell proliferation assay and EBOV infectivity

Proliferation assay was performed using CellTrace CFSE Cell Proliferation Kit (Thermo Fisher Scientific) following the manufacturer’s instructions. Briefly, fresh PBMCs were obtained from healthy donor blood as described above. PBMCs were labeled with CFSE and incubated with 20% FBS to quench excess CFSE and washed three times with PBS. Cells were mock-treated or cultured with VLPs for 48 h at 37°C. Then, cells were harvested and analyzed by flow cytometry for CFSE dilution. To evaluate EBOV infectivity, THP-1 cells were pulsed with medium alone or with VLPs for 24 h. Then, cells were centrifuged for 5 min at 250 × *g*, supernatants were removed, and the cells were cultured with EBOV-GFP at an MOI of 0.1 PFU/cell for an additional 48 h. Cells were harvested, fixed with 4% formaldehyde for 24 h at 4°C, and analyzed for GFP expression by flow cytometry.

### Binding and internalization assays

Human donor PBMCs were isolated with a Histopaque (Sigma-Aldrich) gradient as recommended by the manufacturer. Then, T-lymphocytes and B-lymphocytes were isolated from PBMCs by negative selection using magnetic microbead separation kits (Miltenyi Biotec) to keep CD3^+^ and CD19^+^ cells, respectively. The cells were cultured with fresh medium alone or in the presence of WT, mut 5, or mut 14 EBOV VLP for 2 h on ice. Thereafter, cells were immunostained with rabbit antibodies raised against EBOV virus-like particles (VLP) (Integrated BioTherapeutics) and analyzed by flow cytometry. To evaluate the role of TLR4 in binding and internalization of shed GP, HEK 293-TLR4, or THP-1 cells were plated at 10^6^ cells per well in U-bottom 96-well plates (Thermo Fisher Scientific), mock-treated or pre-treated with rTLR4 (RnD Systems, #1478-TR-050), and placed on ice to prevent internalization of VLPs. EBOV shed GPs were added, and cells were incubated for 2 h on ice and washed with PBS containing 2% heat-inactivated FBS. Then, to investigate internalization, cells were either Immediately trypsinized or incubated at 37°C for 1 h to promote internalization and then trypsinized; non-trypsinized samples were used as a control. Thereafter, cell lysates were immunostained with the following antibodies: anti-EBOV GP (Integrated BioTherapeutics, #0201-020), anti-TLR4 (Santa Cruz Biotechnology, #sc-293072), and anti-GAPDH (Cell Signaling, #8884S).

### Analysis of signaling pathways

Isolated PBMCs were plated at a concentration of 2 × 10^6^ cells per well in 24-well plates (Thermo Fisher Scientific). Then, the cells were stimulated or not with 25 ng/mL TPA (Sigma-Aldrich) and with 0.5 µM ionomycin (Sigma-Aldrich), treated or not with 1 µM of CsA (Sigma-Aldrich), or incubated with WT, mut 5, and mut 14 VLPs for 16 h at 37°C, and washed with PBS containing 2% heat-inactivated FBS. Cell lysates were collected for western blot analysis with antibodies specific for multi-p NFAT1 and mono-p NFAT1 (Novus Biologicals, #25A10.D6.D2), p-AkT (Cell Signaling, #9271), AkT (Cell Signaling, #4691), p-mTOR (Cell Signaling, #2971) and mTOR (Cell Signaling, #2972), EBOV VP40 (Integrated BioTherapeutics, #0301-010), and GAPDH (Cell Signaling, #8884S).

### Imaging flow cytometry analysis

THP-1 cells were transfected with a plasmid coding for NFAT1-GFP protein previously described (73) using Lipofectamine LTX reagent (ThermoFisher Scientific) according to the manufacturer’s instructions. Then, transfected THP-1 cells were cultured at 10^6^ cells per well in a 96-well plate, stimulated or not with TPA (Sigma-Aldrich) at 25 ng/mL and ionomycin at 0.5 µM (Sigma-Aldrich), cultured with medium alone or with VLPs for 16 h, harvested, and stained as described for flow cytometry experiments above. Analyses of EBOV VLP proteins and NFAT1-GFP proteins were performed using an AMNIS FlowSight Imaging flow cytometer (Sigma-Aldrich) with a minimum of 2,000 events acquired. The purity of the virions was monitored and confirmed during imaging flow cytometry experiments with gating by “aspect ratio” on the *y*-axis and “area” on the *x*-axis in the bright-field channel. Data were analyzed with the IDEAS version 2.0 software.

### Luciferase assays

293T and 293-TLR4 cells were seeded at 10^5^ cells per well in 12-well plates (Sigma-Aldrich), transfected with NFκB-Luc (Addgene, #111216) or NFAT-Luc (Addgene, #17870) plasmids using TransIT LT1 transfection reagent (Mirus Bio LLC) and incubated at 37°C for 48 h. Cells were then stimulated with 25 ng/mL TPA and 0.5 µM of ionomycin, or 1 µM of CsA, 10 µg/mL of rTLR4 (RnD Systems, #1478-TR-050), or 100 ng/mL CLI-095 (InvivoGen) for 1 h. Next, cells were pulsed with medium alone or with EBOV VLPs for an additional 24 h. Then, cells were lysed with Pierce Luciferase Cell lysis buffer (Thermo Fisher Scientific), and cell lysates were assayed for luciferase activity using a luminometer (Glomax 20/20, Promega). Bicinchoninic acid (BCA) protein assays (Thermo Scientific) were used for normalization.

### Multiplex analysis of serum cytokines and chemokines

Isolated PBMCs were seeded at 10^6^ cells per ml in a 12-well plate (Corning). Then, cells were cultured with medium alone or in the presence of WT shed GP, mut 5 shed GP, or mut 14 shed GP for 24 h or 96 h. Supernatants were harvested and centrifuged at 9,000 × *g* for 10 min at 4°C to remove cell lysates or debris. Next, supernatants were analyzed using a Multiplex magnetic bead-based assay (Eve Technologies) to evaluate cytokine levels for GM-CSF, IFNγ, IL-6, IL-8, IL-12(p70), TNFα, IL-4, IL-10, IL-5, and IL-13.

### Monocyte adhesion assay

Human brain microvascular endothelial cell (HBMVEC), passages 6 and 7, were seeded on 48-well plates and cultured at 37°C and 5% CO_2_. The traditional fluorescent microscopy-based monocyte adhesion assay was processed as previously described (48). Briefly, calcein-AM-labeled THP-1 monocytes (5 × 10^4^ cells per well) were added to HBMVEC monolayer (5 × 10^4^ cells/well) in 48-well plates. After co-cultures for a designed time, non-adherent monocytes were gently washed off using PBS, and adherent monocytes were fixed with 4% paraformaldehyde. Monocyte adhesion was visualized under a fluorescent microscope (Olympus BX51) using a 10 × objective. The relative fluorescence intensity was calculated using ImageJ software from three different fields per well. The results are representative of at least three independent experiments.

### Fluidic AFM single-living THP-1-HBMVEC vertical binding force (VBF) measurement

For the fluidic AFM studies, the HBMVECs were co-cultured with THP-1 cells at 1:1 ratio. The fluidic AFM system coupling Nanosurf Core AFM (Nanosurf) and Fluidic Pressure Controller (Cytosurge AG) was used for this assay. The air in the reservoir on the backside of the micropipette microchannel was removed by PBS, and the reservoir was connected to the Pneumatic Connector (Cytosurge AG). After subsequent connection to the Fluidic Pressure Controller, positive pressure (20 mBar) was applied to enable PBS to flow through the microchannel within the micropipette. Under a phase contrast microscope, the micropipette Fluidic AFM cantilever (Cytosurge AG) approached a single monocyte in medium, and a −800-mbar pressure was applied to capture a single THP-1 cell by adsorbing it onto the aperture of the micropipette. This micropipette was then used as a cell probe to measure the VBFs between an HBMVEC and the THP-1 cell on the micropipette using force spectroscopy as we described (45). The cell-micropipette was driven to approach the HBMVEC monolayer with 2 nN as the setpoint force and paused on the surface of the cell for 0.5 min. This defined time established the interaction on the surfaces between a single THP-1 and HBMVEC. The force spectroscopy was performed to measure the unbinding force during rupture of the interaction between the THP-1 and HBMVEC. The unbinding effort was assessed by measuring the work done (in picojoules [pJ]), which was calculated by integrating the area under the force-distance (F-D) curve (representative curves shown in Fig. 6F) using software as described previously (47, 74). The Temperature Controller (Nanosurf) kept the liquid environment at 37°C during all measurements. To avoid any cell debris affecting the microfluid channel of the cantilever from previous measurements, a new micropipette was routinely installed for a new measurement. At least five single sets of THP-1–HBMVEC were measured with five cantilevers, respectively, per group (*n* = 5 per group in Fig. 6B and D).

### Statistical analyses

Each independent experiment was performed in biological duplicates or triplicates. Data are presented as mean ± SEM of at least three independent experiments. Statistical methods used are mentioned in figure legends, and the statistics were calculated using GraphPad Prism 6 (GraphPad). *P* values of <0.05 were considered statistically significant.

## SUPPLEMENTAL MATERIAL

Supplemental Figures mbio.02278-25-s0001.pdf Figures S1-S3.

ASM does not own the copyrights to Supplemental Material that may be linked to, or accessed through, an article. The authors have granted ASM a non-exclusive, world-wide license to publish the Supplemental Material files. Please contact the corresponding author directly for reuse.
