# Supplementary material for: Distinct immune properties of the N- and C-termini of the immunosuppressive domain of Ebola virus glycoprotein
Source: mBio. 2025 Oct 9;16(11):e02278-25. doi: 10.1128/mbio.02278-25 (PMC12607705; doi:10.1128/mbio.02278-25)
Supplement: Supplemental Figures — Figures S1-S3. [file mbio.02278-25-s0001.pdf]

A

EBOV GP	ISD Sequences
WT	I L N R K A I D F L L Q R W G G T
mut 1	A L N R K A I D F L L Q R W G G T
mut 2	I A N R K A I D F L L Q R W G G T
mut 3	I L A R K A I D F L L Q R W G G T
mut 4	I L N A K A I D F L L Q R W G G T
mut 5	I L N R A A I D F L L Q R W G G T
mut 6	I L N R K G I D F L L Q R W G G T
mut 7	I L N R K A A D F L L Q R W G G T
mut 8	I L N R K A I A F L L Q R W G G T
mut 9	I L N R K A I D A L L Q R W G G T
mut 10	I L N R K A I D F A L Q R W G G T
mut 11	I L N R K A I D F L A Q R W G G T
mut 12	I L N R K A I D F L L A R W G G T
mut 13	I L N R K A I D F L L Q A W G G T
mut 14	I L N R K A I D F L L Q R A G G T
mut 15	I L N R K A I D F L L Q R W A G T
mut 16	I L N R K A I D F L L Q R W G A T
mut 17	I L N R K A I D F L L Q R W G G A

B

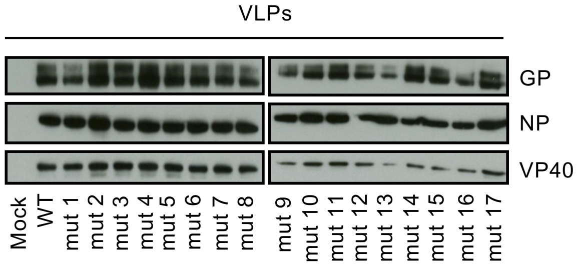

Suppl. Fig. 1. Production of EBOV VLPs with mutated ISD on GP.
A. Mutations in the VLP constructs indicated in red.
B. Western blot analysis of VLPs harvested from supernatants following transfection of 293T cells with plasmids coding for NP, VP40 and each GP construct.

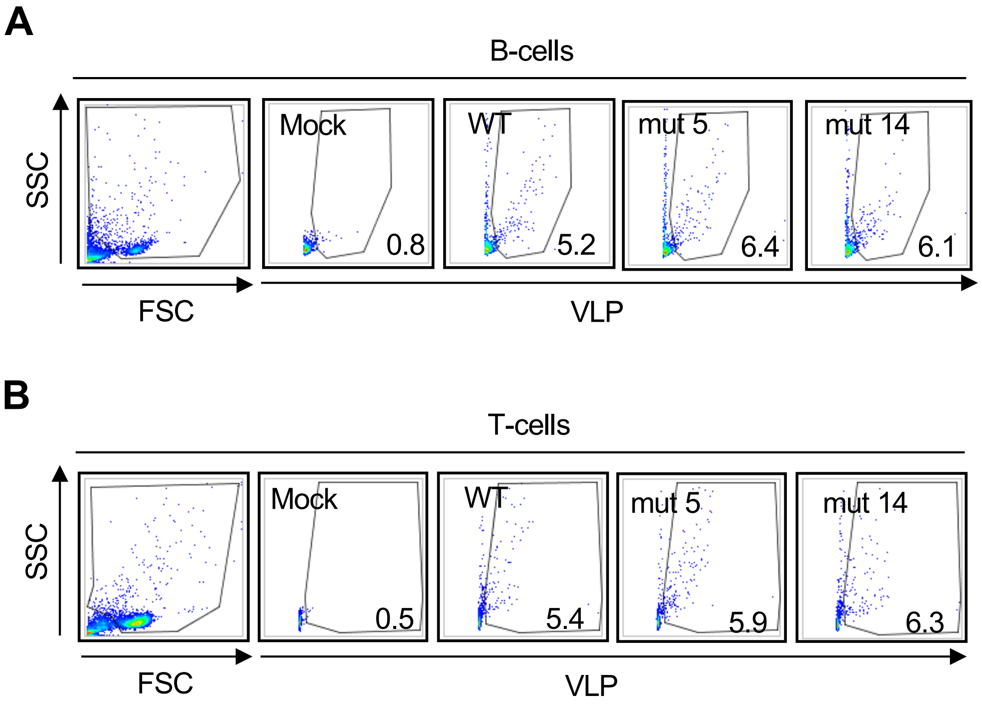

Suppl. Fig. 2. Mut 5 and mut 14 EBOV ISD do not impair EBOV binding to B- and T-cells.
Flow cytometry analysis of WT, mut 5 and mut 14 EBOV VLPs binding to B-cells (**A**) and T-cells (**B**). Data are representative from 3 independent experiments.

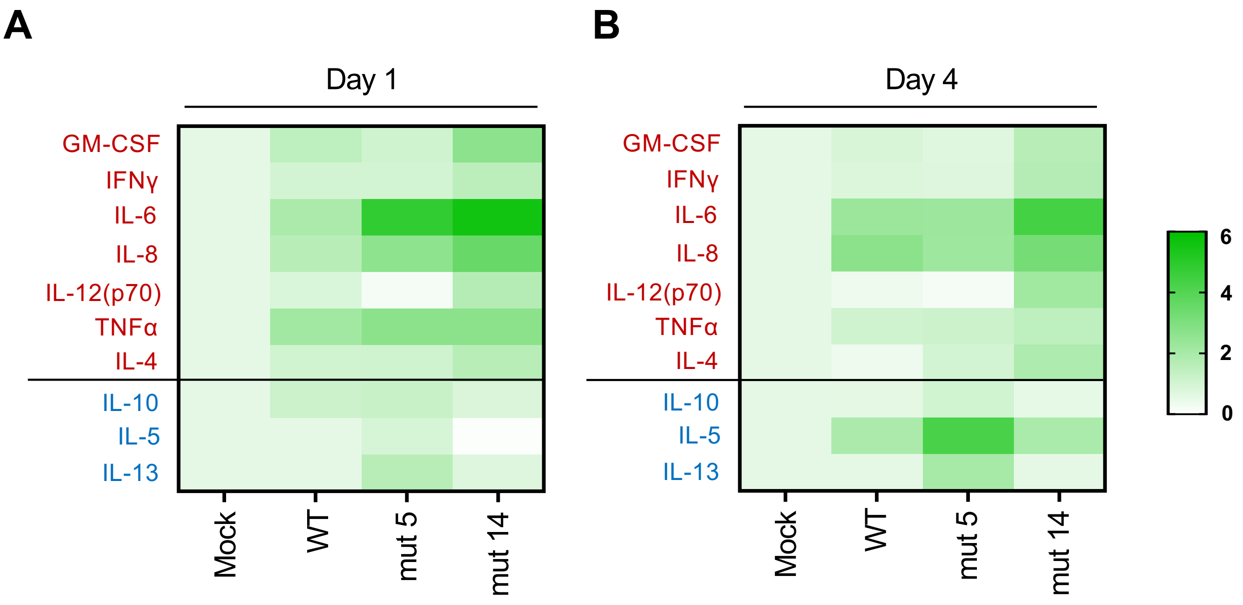

Suppl. Fig. 3. N- and C-termini of EBOV shed GP ISD differentially trigger the expression of pro- and anti-inflammatory cytokines.

Multiplex assay quantifying pro-inflammatory cytokines (indicated in dark-red) and anti-inflammatory cytokines (indicated in blue). PBMCs were cultured with WT, mut 5 and mut 14 EBOV shed GP or medium for 24 h (**A**) or 96 h (**B**). The values were normalized on mock-treated cultures. Unlike Fig. 7, the data are normalized to the samples which were incubated without shed GP. Representative data from at least 3 experiments with PBMCs from individual donors.
